# Auditory repetition suppression alterations in relation to cognitive functioning in fragile X syndrome: a combined EEG and machine learning approach

**DOI:** 10.1186/s11689-018-9223-3

**Published:** 2018-01-29

**Authors:** Inga Sophia Knoth, Tarek Lajnef, Simon Rigoulot, Karine Lacourse, Phetsamone Vannasing, Jacques L. Michaud, Sébastien Jacquemont, Philippe Major, Karim Jerbi, Sarah Lippé

**Affiliations:** 1 Neuroscience of Early Development (NED), 90 Avenue Vincent-D’indy, Montreal, QC H2V 2S9 Canada; 2 Research Center of the CHU Sainte-Justine Mother and Child University Hospital Center, 3175 Chemin Côte Ste-Catherine, Montreal, QC H3T 1C5 Canada; 3 Department of Psychology, Université de Montréal, 90 Avenue Vincent-D’indy, Montreal, QC H2V 2S9 Canada; 4 Centre de Recherche en Neuropsychologie et Cognition (CERNEC), 90 Avenue Vincent-D’indy, Montreal, QC H2V 2S9 Canada; 5 International Laboratory for Brain, Music and Sound Research (BRAMS), 1430 Boul Mont-Royal, Montreal, QC H2V 2J2 Canada; 6 Faculty of Medicine, Université de Montréal, 2900 boulevard Édouard-Montpetit, Montréal, QC H3T 1J4 Canada; 7 Centre de Recherche de l’Institut Universitaire en Santé Mentale de Montréal (CRIUSMM), 7401 Rue Hochelaga, Montréal, QC H1N 3M5 Canada; 8 Centre de Recherche de l’Institut Universitaire de Gériatrie de Montréal (CRIUGM), 4565, chemin Queen-Mary, Montreal, QC H3W 1W5 Canada

**Keywords:** Fragile X syndrome, Intellectual disability, EEG, Repetition suppression, Machine learning, Habituation, IQ, Cognition

## Abstract

**Background:**

Fragile X syndrome (FXS) is a neurodevelopmental genetic disorder causing cognitive and behavioural deficits. Repetition suppression (RS), a learning phenomenon in which stimulus repetitions result in diminished brain activity, has been found to be impaired in FXS. Alterations in RS have been associated with behavioural problems in FXS; however, relations between RS and intellectual functioning have not yet been elucidated.

**Methods:**

EEG was recorded in 14 FXS participants and 25 neurotypical controls during an auditory habituation paradigm using repeatedly presented pseudowords. Non-phased locked signal energy was compared across presentations and between groups using linear mixed models (LMMs) in order to investigate RS effects across repetitions and brain areas and a possible relation to non-verbal IQ (NVIQ) in FXS. In addition, we explored group differences according to NVIQ and we probed the feasibility of training a support vector machine to predict cognitive functioning levels across FXS participants based on single-trial RS features.

**Results:**

LMM analyses showed that repetition effects differ between groups (FXS vs. controls) as well as with respect to NVIQ in FXS. When exploring group differences in RS patterns, we found that neurotypical controls revealed the expected pattern of RS between the first and second presentations of a pseudoword. More importantly, while FXS participants in the ≤ 42 NVIQ group showed no RS, the > 42 NVIQ group showed a delayed RS response after several presentations. Concordantly, single-trial estimates of repetition effects over the first four repetitions provided the highest decoding accuracies in the classification between the FXS participant groups.

**Conclusion:**

Electrophysiological measures of repetition effects provide a non-invasive and unbiased measure of brain responses sensitive to cognitive functioning levels, which may be useful for clinical trials in FXS.

**Electronic supplementary material:**

The online version of this article (10.1186/s11689-018-9223-3) contains supplementary material, which is available to authorized users.

## Background

Fragile X Syndrome (FXS) is a neurodevelopmental genetic disorder, which causes cognitive and behavioural deficits. FXS is caused by a mutation of the *FMR1* (‘fragile X mental retardation 1’) gene located on the X chromosome [1] that prevents expression of the fragile X mental retardation protein (FMRP) [2]. The majority of individuals affected by FXS have an intellectual disability (ID), ranging from mild to severe in males and from mild to moderate in females [3]. Cognitive impairments are often found in language, executive functions and visuo-spatial and social-cognitive domains [4]. Particular impairments are found in auditory working memory span and working memory for words [5]. Many of the symptoms found in FXS are typical of autistic spectrum disorders (ASD), [6] including aberrant behaviours, emotional instability and hyperarousal to sensory stimulation [4], especially in the auditory modality [7].

Deficits in auditory processing likely contribute to behavioural hypersensitivity and hyperexcitability to auditory stimulation reported in FXS individuals [8–10] and may be involved in abnormal language development as suggested by studies investigating autism [7, 11, 12]. Electroencephalography (EEG) studies revealed alterations in auditory evoked potentials (AEPs) reflecting basic auditory processing deficits in FXS [13–18]. These deficits are perhaps impairing the generation of memory traces, a concept reflecting the memorization of a learnt stimulus, which is required for stimulus discrimination [18] and may thus be related to a lack of habituation. EEG alterations in FXS have been found not only in basic auditory processing, but also in later event-related potential (ERP) components reflecting cognitive processes. A classic protocol to elicit cognitive ERPs is the oddball paradigm: trains of frequent standard stimuli are randomly interspersed with rare deviant stimuli eliciting a particular response, such as the Mismatch Negativity (MMN) in passive tasks and the P3 in active tasks in which a response to the infrequent stimuli is required [19]. Amplitudes of MMN and P3 components are attenuated in FXS [16–18], suggesting poor memory trace formation of the standard stimulus [20] as well as attention deficits [21].

Repetition suppression (RS) describes a phenomenon in which stimulus repetitions result in diminished brain activity in response to the standard stimulus. Using EEG, auditory RS in FXS has been assessed by comparing responses to early and late standard tones in oddball paradigms [15, 18] and by analysing a maximum of four sequential presentations of a standard tone [9, 13]. Both paradigms consistently show a lack of N1/P2 amplitude suppression in FXS. Recently, Ethridge et al. [9] analysed single-trial time–frequency in addition to AEP habituation and reported a decrease of RS in N1 amplitude together with alterations in both power and phase locking index in several frequency bands in FXS [9].

Whereas impairments in RS have been repeatedly found in FXS [9, 13, 15, 18, 22, 23], it has not yet been investigated with regard to cognitive functioning. In an exploratory analysis, Ethridge et al. suggested that reductions in RS were associated with parental reports of auditory hypersensitivity and social problems in FXS participants [9]. In support of these findings, Bruno et al. found impairments in RS to be correlated to higher autism symptoms in FXS [22]. However, no reference to ID severity has been made. Given that RS appears to be associated with auditory perceptual learning [24] and learning being a prerequisite for cognitive functioning, we expect RS patterns to vary in FXS with regard to IQ. Further, habituation, the behavioural pendant to repetition suppression, has been found to predict later IQ in infant populations [25], suggesting a possible link between RS and IQ.

In order to reveal the distinct patterns of repetition effects in FXS participants in relation to cognition, we used a passive listening paradigm presenting ten standards without deviants, allowing measurement of a delayed repetition effect with more repetitions. In addition, we extended the investigation of RS from basic sensory components, as performed previously, to stimuli mimicking words. Processing of such stimuli is typically reflected in early as well as late components such as the N400 [26]. In fact, loss of N400 RS in response to spoken target words was found in fragile X-associated tremor/ataxia syndrome (FXTAS) [27]. We aimed at controlling for familiarity by using novel complex auditory stimuli in order to avoid semantic information that might bias cerebral processing and elicit late cognitive components such as the N400 [2, 28].

Auditory RS can also be studied in FXS animal models [29] supporting the relevance of RS as a translational biomarker for therapeutic approaches [30, 31]. Lovelace et al. demonstrated that a class of enzymes targeted by FMRP is directly involved in RS in *FMR1* KO mice [29]. In line with this, Bruno and colleagues [22] used fMRI and found impaired RS to visual face/gaze stimuli in the left fusiform gyrus directly correlated to lower, less typical levels of FMRP in FXS participants. Importantly, Schneider et al. used RS as an outcome measure in a clinical trial and found an improvement of RS in the N1/P2 complex in response to late vs. early sinusoidal tones in FXS participants after 3 months of minocycline treatment [15], pointing to the possibility of rescuing RS in humans as it was found in *FMR1* KO mice [29]. To further explore the clinical potential of this measure, we also used a machine learning approach to quantify the accuracy of single-trial RS features in the prediction of cognitive functioning levels in FXS participants.

## Methods

### Participants

A total of 19 FXS participants and 29 neurotypical controls participated in the experiment. Five FXS participants and three controls were excluded from analysis due to extensive movement artifacts. The 14 remaining FXS participants were compared to 26 neurotypical controls with a similar age distribution. Table 1 displays the demographics of the study population. Medication was reported by the parents, and all FXS participants were on a stable dose since at least 6 months before testing. Diagnoses of comorbidities were obtained from the medical file at the hospital and were based on psychiatric and/or neuropsychological evaluations. Medication and comorbidities are detailed in Table 2.

**Table 1:** Demographics of the study population

Variable	FXS participants	Neurotypical controls
*N*	14 (4♀)	26 (11♀)
Age range	9–32 years	9–32 years
Mean age (SD)	15.5 (± 6.06)	17.1 (± 6.1)
NVIQ range	32–93	87–129
Mean NVIQ (SD)	48 (± 14.12)	113 (± 10.41)

**Table 2:** Characteristics of the FXS NVIQ median-split subgroups

Variable	≤ 42 NVIQ group	> 42 NVIQ group
*N*	8 (0 female)	6 (4 female)
Age range	9–32 years	10–22 years
Mean age (SD)	16.38 (± 7.37)	14.34 (± 4.08)
NVIQ range	32–42	52–93
Mean NVIQ (SD)	38 (± 3.64)	62 (± 10.02)
Medication	*N* = 6 Methylphenidate (36–45 mg qd) (4) Amphetamine mixed salts (50 mg qd) (1) Venlafaxine (75 mg qd) (1)	*N* = 3 Methylphenidate (36–50 mg qd) (2) Atomoxetine (25–40 mg qd) (2) Citalopram (20 mg qd) (1)
Comorbidities	Autistic spectrum disorder (4) Attention-deficit hyperactivity disorder (6)	Autistic spectrum disorders (1) Attention deficit hyperactivity disorder (3)

Patient recruitment was based on DNA analyses previously conducted in the genetics department of the CHU Sainte-Justine Mother and Child University Hospital Center in Montreal. Neurotypical controls were recruited using posters at the Sainte-Justine Hospital and the University of Montreal and by classified ads on selected websites. Normal hearing and normal or corrected-to-normal vision was reported in all participants. All participants were francophone, right-handed and born at term. Non-verbal intelligence was examined using the non-verbal Leiter-R International Performance Scale [32] for all FXS participants as well as neurotypical children and adolescents and the Wechsler Abbreviated Scale of Intelligence (WASI) [33] for neurotypical adults only. Autistic behaviour in FXS participants was quantified using the repetitive behavior questionnaire [34] and the aberrant behavior checklist [35], which were completed by the parents. Results from these questionnaires are reported in [14]. The study protocol was reviewed and approved by the ethics, administrative and scientific committees at the Sainte-Justine’s Hospital Research Center. Procedures undertaken were explained to participants and parents or legal caregivers, and written informed consent was obtained.

### Apparatus

Testing took place in a dark soundproof experimental chamber in the Sainte-Justine hospital. A Dell GX150 PC was used to present the stimuli via E-Prime 1.0 (Psychology Software Tools Inc. Pittsburgh, PA, USA). Two speakers (Optimus XTS 24, Boston, MA, USA) were placed laterally at a 30-cm distance from the subject’s ears.

### Stimuli

Eighteen different two-syllable pseudowords were chosen from the BELEC [36] and ODÉDYS-II [37] paediatric batteries and recorded in a soundproof chamber while spoken by a native French-speaking woman. Adobe Audition 3.0 (Adobe Systems Inc., San Jose, CA, USA) was used for recording and normalization to − 3 dB SPL. Pseudowords had an average length of 930 ms and ranged between 800 and 1300 ms.

### Procedure

Participants chose among five movies for children that they watched without sound and without subtitles during EEG installation and stimuli presentation in order to enhance acceptance of the procedure and reduce movement artefacts through fixation on the screen. The same pseudoword was presented successively ten times each with an inter-stimulus interval of 250 ms at 70 dB SPL intensity and 16-bit resolution. In total, 18 trials with different pseudowords were presented in sequential order with an inter-trial interval of 250 ms in a passive listening paradigm. The order of pseudowords was fixed across participants in order to avoid pseudowords starting with a similar sound to be presented in succession.

A 128 electrode dense array EEG was used for recording (Electrical Geodesics System Inc., Eugene, OR, USA). Impedances were maintained under 40 kΩ [38], and during recording, Cz was used as reference. Signals were acquired and processed by a G4 Macintosh computer using NetStation Software (Version 2.0). EEG data was digitalized at a sampling rate of 250 Hz, and an analog bandpass filter of 0.01–100 Hz was applied. Off-line analyses were carried out with BrainVision Analyser software, version 2.0 (Brain Products, Munich, Germany). Data were digitally filtered with a 1–50 Hz filter and re-referenced to an average reference. Thirty electrodes containing muscular artefacts, around the neck and face, were removed for all participants. Blink artefacts were removed using a semi-automatic independent component analysis (ICA) [39] (see Additional file 1 for details). EEG signal was segmented into 800-ms epochs after stimulus onset. Algorithmic artefact marking of voltage exceeding ± 100 μV was followed by visual data inspection of segmented data in which epochs with artefacts were rejected manually. An average of 178/180 segments were kept for control participants and 174/180 for FXS participants*.*

### EEG signal processing

Data was exported to a commercial software package (MATLAB 6.1, The MathWorks Inc., Natick, MA, 2000) using BrainVision solutions. Signal energy (*E*) was used as a measure of total signal amplitude in order to detect repetition effects as in [40]. Signal energy measures allowed the inclusion of a larger age range compared to ERP component analyses, since it is less affected by maturational changes found in AEP components [41].

Signal energy is defined as *E* =  ∑ |*amp*|2 where amp is the amplitude value (μV) of all EEG data points contained in a segmented trial. The time series of each presentation *p* (1 to 10) were normalized relative to the standard deviation of its series of ten presentations (the repetition series) of the pseudoword stimulus *s* (1 to 18) for each participant and channel. The objective was to normalize the time series of each pseudoword *s* (the series of ten presentations of *s*) to make the standard deviation of each pseudoword the same. Subsequently, the energy was computed for all ten presentations (1 ≤ *n* ≤ 10) of pseudowords. A detailed explanation of the signal energy computation can be found in the Additional file 1.

### Spatial principal component analysis

In order to identify spatial regions of interest (ROIs) relevant for the task performed in our samples, we used the properties of principal component analysis (PCA) [42–45]. For each group of participants (FXS participants and controls), we performed a separate spatial PCA (Varimax rotation, SPSS statistics, version 20, IBM Corp., Armonk, NY, USA) with 99 electrode sites as dependent variables, participants (14 in the FXS and 26 in the control group) and presentations (10) as observations [46]. Specific details of the spatial PCA factor loadings can be found in Additional file 1.

**Fig. 1:**
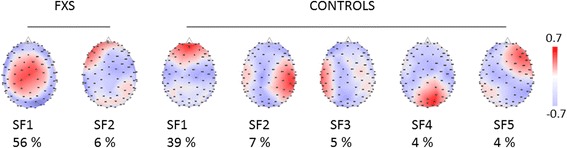
Spatial factors constituting ROIs yielded by PCA explaining > 60% of the data variance in each group (two factors for FXS participants and five factors for the control group)

The spatial PCA yielded 12 factors for the FXS and 15 for the control group. The first five factors explained 60% of the data variance for the control group, and the first two factors explained 62% of the variance for the FXS group. Seven regions of interest were identified from these factors (see Fig. 1): central and left frontal areas based on the two factors for the FXS group and left temporal, fronto-central, right frontal, right temporal and occipital areas based on the five factors for the control group.

### Statistical analysis

#### Linear mixed models

Statistical analyses were performed using SPSS statistics, version 23 (IBM Corp., Armonk, NY, USA). Linear mixed models (LMM) were used to understand how group membership (FXS vs. control) and NVIQ predicted signal energy changes across repetitions. Further, age was assessed as predictor in order to account for the large age range in our sample. A LMM approach was chosen over traditional repeated measures analysis in order to account for unbalanced design, enable random intercepts and slopes, allow for nonlinear modeling of energy changes across repetitions and select an appropriate covariance structure for the repetition effects [47–50].

The model used for this study was determined by a series of steps to determine model fit [47] that can be found in Additional file 1. Finally, LMM analysis was performed using maximum likelihood for estimation method [47] and predictors group, NVIQ and age were added sequentially, verifying if model fit was improved by the addition of each predictor using chi-square likelihood ratio test [47]. Finally, covariance structure was selected by comparing model fit between available structures using Akaike’s Information Criterion (AIC) [47].

Based on significant interactions, further LMMs were performed, exploring signal energy changes across presentations in FXS and controls separately following the same series of steps described above for each model. Bonferroni-corrected post hoc paired comparisons were performed for significant main effects. Significance level was set to 5% (*p* = 0.05). In order to explore significant interactions and reveal patterns of RS, a NVIQ median-split was performed dividing the FXS group into two subgroups (≤ 42 and > 42 NVIQ).

### Classification of NVIQ subgroups in FXS using single-trial RS features

A machine learning approach was used in order to specifically investigate whether differences in RS effects can predict differences in cognitive capacities (i.e. NVIQ levels) within the FXS group. Importantly, we chose to explore this question using a binary classification approach (to decode between NVIQ median-split subgroups) based on single-trial differences in EEG energy between consecutive presentations (18 trials across 14 FXS participants, i.e. *n* = 252). In addition to addressing the limitation of small sample size, which precludes a standard statistical analysis, a successful single-trial classification of FXS participants (i.e. based on 252 samples) would provide an important demonstration of the sensitivity of RS. The features used in the classification consisted of differences in energy between P1 and subsequent presentations as well as differences between P2 and subsequent presentations. Because they capture single-trial changes in energy between first (or second) stimulus presentations and subsequent presentations, these features were designed to account for repetition effects. A total of 17 such features were calculated for each of the seven ROIs, yielding 119 features in total.

We ran the single-trial classifications using a leave-two-subject-out cross-validation across the group of FXS participants, which is equivalent to a K-fold cross-validation where all 36 trials from two participants (one from each subgroup) are used as test set in each fold. Given that the NVIQ based division of the FXS group yielded a subgroup with NVIQ ≤ 42 (*n* = 8) and another with NVIQ > 42 (*n* = 6), we used a bootstrap approach to repeatedly run the classification on balanced classes. This led to running 28 classifications (all options of picking subgroups of 6 among 8 participants) with 216 samples (6 × 18 trials). In other words, in each fold, a model is trained on single-trial RS features from ten participants (5 per NVIQ subgroup) and tested on the single trials from the two remaining participants (1 from each class). The mean decoding accuracy (DA) of each single feature was used as a measure of classification performance.

Several classification algorithms were tested including *k*-th nearest neighbor (KNN), linear discriminant analysis (LDA) and support vector machine (SVM). Although the performances were reasonably similar, SVM (with radial basis function kernel) provided the best decoding results and was thus used in this study.

Given that the decoding problem investigated here is a binary classification, the theoretical chance level for the DA is 50%. However, a reliable assessment of the accuracy of machine learning decoding accuracy requires an evaluation in terms of statistical significance. We therefore evaluated the statistical significance of all reported DAs using the binomial cumulative distribution [51], followed by Bonferroni correction across the number of explored features to correct for multiple comparisons. This conservative approach indicated that a decoding accuracy is considered statistically significant at *p* < 0.05 or *p* < 0.01 if it exceeds 62.96 or 64.35% respectively.

## Results

### Characteristics of the population

FXS participants had a lower NVIQ (*M* = 51, ± 15.46) than the control group (*M* = 113, ± 10.79) (*t*_(23)_ = − 15.5, *p* = 0.0001). Based on our NVIQ measures, one participant (14 years, NVIQ = 93, female) did not present an ID. The rest presented an ID ranging from mild to severe. For some analyses, the FXS group was split into subgroups using a median-split at 42 NVIQ. Characteristics of the NVIQ-FXS subgroups can be found in Table 2. NVIQ differed significantly between the two FXS subgroups (*t*_(12)_ = − 5.6, *p* = 0.001) with a mean of 38 (± 3.64) vs. 62 (± 10.02). EEG segments kept for analysis did not differ significantly between control and FXS participants (*t*_(15)_ = − 2.1, *p* = 0.058) or between the two NVIQ FXS subgroups (*t*_(8)_ = − 1.9, *p* = 0.099).

### Linear mixed models

#### Baseline model: intercept, slope and polynomial structure

The construction of the model was started with a simple repeated measures (repetition (10) × ROI (7)) model with energy as outcome variable and repetition and ROI as fixed effects and without any predictors that served as baseline model. Using the chi-square likelihood ratio test, best fit for the baseline model was found using a random slope but not intercept and a linear model (see Additional file 1 for details).

### Predictors

The first predictor added to the model was group (FXS vs. control) in order to verify if information about group membership improves model fit. Repetition, ROI and group were entered as fixed effects as well as interactions between repetition and ROI; repetition and group; and repetition, group and ROI. A random slope term accounted for inter-individual differences in trajectory changes across repetitions. Adding the predictor ‘group’ improved the model significantly [*χ*^2^ (70, *N* = 40) = 103, *p* < 0.01]. Whereas no significant main effect was found for group and ROI, repetition yielded a significant effect (*F* (9, 395.4) = 5.77, *p* = 0.0001), meaning that signal energy significantly changed across stimulus repetitions. A significant interaction was found between repetition and group (*F* (9, 395.4) = 3.75, *p* = 0.0001), suggesting that signal energy repetition effects differed between groups. No interactions were found between ROI and repetition or ROI, group and repetition.

Then, NVIQ was added as a second predictor and fixed effect to the model. Interactions between NVIQ and repetition as well as between NVIQ, repetition and group were added to the existing interactions. The model improved significantly [*χ*^2^ (20, *N* = 40) = 43, *p* < 0.01] with the inclusion of the predictor ‘NVIQ’. In this model, the main effect for repetition was not significant any more (*F* (9, 596) = 1.43, *p* = 0.174) and neither were the other main effects (ROI, IQ, group). A significant interaction was found between repetition and group (*F* (9, 596) = 2.09, *p* = 0.029), as well as between repetition and NVIQ (*F* (9, 602) = 2.05, *p* = 0.032) and repetition, NVIQ and group (*F* (10, 417) = 2.57, *p* = 0.005), suggesting that repetition effect differences between groups varied with NVIQ.

Finally, age was added as predictor and fixed effect to the model. Adding age as predictor diminished model fit according to AIC and differed not significantly from the previous model [*χ*^2^ (40, *N* = 40) = 41, *p* < 0.9].

Thus, we concluded that a random slope model with group and NVIQ as predictors presents the best fit for the data. All available covariance structures were tested, and first-order autoregressive covariance structure provided the best model fit according to AIC. Based on the significant interactions between group and repetitions, we decided to build separate models for FXS and controls in order to examine their distinct repetition effect patterns.

### Controls

**Fig. 2:**
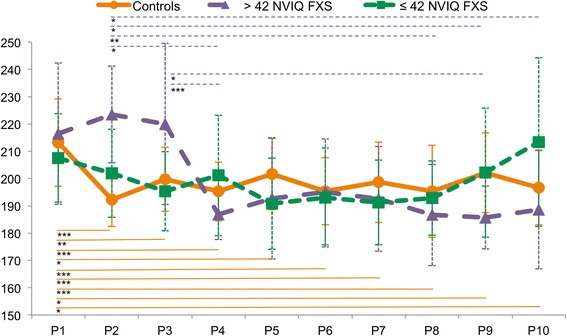
EEG signal energy across presentations one through ten (P1–P10) over all ROIs averaged in the control group and the ≤ 42 and > 42 NVIQ FXS subgroups. Error bars are showing standard deviations. ***p < 0.001, **p < 0.01, *p < 0.05

The test statistics for the baseline model can be found in Additional file 1. A significant main effect was found for repetition (*F* (9, 273.3) = 9.31, *p* = 0.0001), meaning that signal energy significantly changed between repetitions, but not for ROI. A significant interaction was found between ROI and repetition (*F* (54, 1253) = 1.52, *p* = 0.01). A Bonferroni-corrected post hoc test showed a significant reduction in energy between the first and all following presentations of a pseudoword (see Table 3 for mean values and *t* statistics). Figure 2 shows energy across presentations for the control group. The addition of NVIQ as predictor did not improve the model significantly [*χ*^2^ (10, *N* = 26) = 15, *p* > 0.05].

**Table 3:** Mean energy (±SD) for each presentation and participant group and *t* statistics for significant energy differences between presentations (Bonferroni corrected *p*-values for multiple comparisons)

Presentations	Controls	FXS ≤ 42 NVIQ	FXS > 42 NVIQ
1	213.2 (± 16)	207.6 (± 16.2)	216.5 (± 25.9)
2	192.2 (± 9.8)	202 (± 16.1)	223.5 (± 17.7)
1 vs. 2	*t*_(471)_ = 7.5, *p* = 0.0001		
3	199.8 (± 11.8)	195.3 (± 14.5)	220 (± 29.5)
1 vs. 3	*t*_(304)_ = 4.2, *p* = 0.001		
4	195.3 (± 10.8)	201.2 (± 22.1)	186.9 (± 8.2)
1 vs. 4	*t*_(258)_ = 5.6, *p* = 0.0001		
2 vs. 4			*t*_(74)_ = 5.2, *p* = 0.004
3 vs. 4			*t*_(101)_ = 2.7, *p* = 0.0001
5	201.6 (± 10.8)	190.8 (± 16.7)	192.7 (± 22.2)
1 vs. 5	*t*_(249)_ = 3.6, *p* = 0.013		
6	195.4 (± 12.3)	193.1 (± 18.1)	195.1 (± 19.4)
1 vs. 6	*t*_(247)_ = 5.5, *p* = 0.0001		
7	198.7 (± 14.7)	191.3 (± 15.5)	192.6 (± 19.2)
1 vs. 7	*t*_(247)_ = 4.5, *p* = 0.0001		
8	195.3 (± 16.9)	192.9 (± 13.7)	186.7 (± 18.6)
1 vs. 8	*t*_(247)_ = 5.6, *p* = 0.0001		
2 vs. 8			*t*_(44)_ = 2.8, *p* = 0.009
9	202.1 (± 14.6)	202.2 (± 23.7)	185.7 (± 11.5)
1 vs. 9	*t*_(247)_ = 3.4, *p* = 0.023		
2 vs. 9			*t*_(42)_ = 3.7, *p* = 0.015
3 vs. 9			*t*_(43)_ = 2.4, *p* = 0.043
10	196.7 (± 13.7)	213.4 (± 30.9)	188.7 (± 21.7)
1 vs. 10	*t*_(247)_ = 5.1, *p* = 0.0001		
2 vs. 10			*t*_(42)_ = 2.3, *p* = 0.038

### FXS

The baseline model is described in Additional file 1. In the baseline model, with fixed effects for repetition, ROI and the interaction between repetition and ROI, a significant effect could be found for repetition (*F* (9, 133.5) = 2.02, *p* = 0.042), meaning that signal energy changed significantly between repetitions. No main effect could be found for ROI, and the interaction between repetition and ROI was also not found to be significant.

Then, we added NVIQ as predictor and fixed effect to the model, as well as interactions between repetition and NVIQ. The model improved significantly with the addition of NVIQ as a predictor [*χ*^2^ (10, *N* = 14) = 20, *p* < 0.05]. A main effect for repetition was found (*F* (9, 207) = 1.99, *p* = 0.042), but not for ROI or NVIQ. The interaction between repetition and NVIQ was found to be significant (*F* (9, 216.6) = 2.36, *p* = 0.015), suggesting that repetition effects in signal energy differed with respect to NVIQ. Bonferroni-corrected post hoc tests showed no significant changes in energy between the ten presentations. In order to explore the significant interaction between repetition and NVIQ, we decided to split the FXS group into subgroups using a median-split at 42 NVIQ.

### > 42 NVIQ FXS subgroup

The baseline model is detailed in Additional file 1. A significant main effect was found for repetition (*F* (9, 58.9) = 3.76, *p* = 0.001), meaning signal energy changed between repetitions. No main effect was found for ROI or the interaction between ROI and repetition. Bonferroni-corrected post hoc tests showed a significant reduction in energy between presentation 2/3 and later presentations. Test statistics can be found in Table 3. Figure 2 shows energy across presentations for the > 42 NVIQ subgroup.

### ≤ 42 NVIQ FXS subgroup

No significant main effect was found for repetition or ROI, and the interaction between ROI and repetition was not significant. Energy across presentations for the **≤** NVIQ FXS subgroup is illustrated in Fig. 2. Signal energy did not change significantly between repetitions in the ≤ 42 NVIQ FXS group.

### Single-trial RS classification results: NVIQ FXS subgroups

**Fig. 3:**
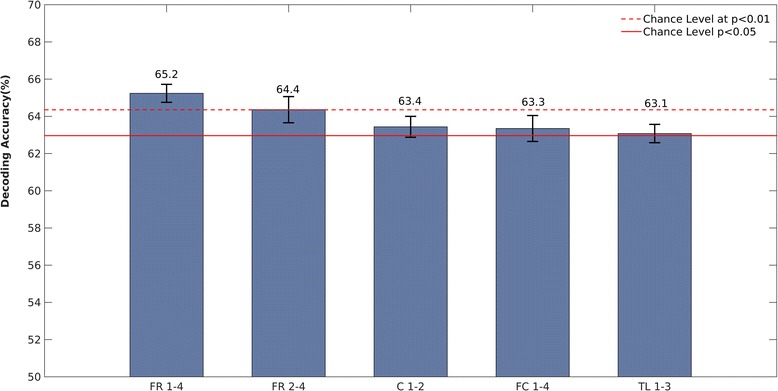
Single-trial SVM classification performance for ≤ 42 vs. > 42 NVIQ FXS subgroups. Each bar represents the percent correct classification achieved with each feature. The features represented on the *x*-axis are single-trial repetition suppression-induced EEG energy modulations between two presentations of the same stimulus, computed within a 0- to 800-ms window (total number of observations for each feature *n* = 252, but 216 were used to ensure balanced classes using bootstrapping; see the ‘Methods’ section for details). The highest decoding (65.2%) was found with FR 1–4, i.e. energy at the right frontal region between presentations P1 and P4. The *y*-axis starts at the theoretical chance level of 50%. The horizontal lines represent respectively (from bottom to top) the chance levels using binomial cumulative distribution for *p* < 0.05 and *p* < 0.01, corrected for multiple comparisons across all 119 features. The error bars represent the standard error on the mean (s.e.m) computed across the bootstrap repetitions. C central, FC fronto-central, FR frontal-right, TL temporal-left

Training an SVM to classify ≤ 42 NVIQ FXS vs. > 42 NVIQ FXS participants based on single-trial EEG repetition effects yielded significant decoding accuracies across four ROIs, mainly over frontal and central regions (Fig. 3). The best predictions of FXS subgroup based on the EEG single-trial data, in other words the highest decoding accuracy, was observed over the frontal-right ROI. More precisely, this was achieved using single-trial RS changes observed between the first and fourth presentations (FR 1–4), yielding 65.2% correct classification, and also between the second and fourth presentations (FR 2–4), with 64.4% correct classifications. The other features that provided statistically significant decoding were obtained with RS measured in the following three ROIs: C 1–2, FC 1–4 and TL 1–3.

## Discussion

In this study, we confirm alterations in the repetition effect brain responses of FXS patients. Differences in repetition effects according to NVIQ in FXS participants were demonstrated for the first time. Neurotypical controls showed the expected pattern of RS between the first and second presentations of a pseudoword and a stable response to subsequent presentations. In FXS participants, NVIQ was a significant predictor of RS patterns. When further exploring this result by separating FXS patients into two groups, we observed RS after four repetitions of a pseudoword in the > 42 NVIQ group, whereas no RS could be found in the FXS participants presenting more cognitive impairment according to their NVIQ scores (≤ 42 NVIQ). Our single-trial machine learning approach further revealed that repetition effects can accurately categorize FXS participants according to their level of cognitive functioning.

### RS in FXS with relation to NVIQ

In controls, RS was found between the first and second presentations of a pseudoword with signal energy remaining stable and low once RS occurred (see Fig. 2). This is in accordance with existing literature stating that RS effects in neurotypical subjects usually happen between the first and second presentations of an auditory stimulus [52]. In FXS, however, the presence of RS varied in relation to NVIQ. Whereas our results in the ≤ 42 NVIQ subgroup replicate findings of no RS in FXS in the literature [9, 13, 15, 18], we are the first to demonstrate a pattern of delayed RS in an FXS full mutation subgroup with a milder cognitive phenotype on average. The fact that RS occurs, although after several repetitions, may be an important building block of cognitive development of these FXS participants, leading to a comparatively milder phenotype. Repetition suppression is suggested to be the electrophysiological signature of habituation [53]. Since habituation has been related to a later cognitive development [25, 54–56], RS measures may be a useful predictor of cognitive phenotype.

The relation of RS with auditory hypersensitivity, social problems [9], autism symptoms and FMRP levels [22] in FXS individuals and animal models [29] has been previously reported. Our findings add the factor of cognitive functioning to the existing literature, further underlining its importance as a sensitive and translational [29] biomarker that could be integrated as an outcome measure in clinical trial protocols [15]. To ascertain the usefulness of repetition effects in a clinical setting or a clinical trial, the consistency of the effects has to be high. Hence, by using a machine learning approach, we were able to reveal the significance and the accuracy rate of the differences in repetition effects with regard to cognitive phenotype. Using a single trial approach allowed us to perform classification on a larger sample size (*n* = 252), but most importantly, it helped demonstrate the consistency of the phenomenon at each trial. The SVM results demonstrate a statistically significant decoding rate (*p* < 10^−5^) when classifying FXS participants according to their NVIQ with more than 65% accuracy using the difference in energy between the four first trials over fronto-central regions. These results are in agreement with the number of repetitions involved in the repetition effects in FXS participants, as revealed by our mixed linear model analyses. Additionally, although based on single-trial training and testing, the cross-validation scheme applied here (cf. the ‘Methods’ section) ensured a strict separation of participants across testing and training conditions. In other words, our machine learning findings reveal the feasibility of classifier generalization across participants with single-trial RS training and testing. These findings may suggest that the proposed method may be used to train a model for fast predictions (e.g. a few trials) in totally naïve FXS participants in particular with larger training sets. In general, our classification results confirm the distinct patterns of repetition effects in the FXS cognitive level subgroups and reveal the potential of our measure in a clinical setting.

### Mechanisms underlying impaired RS in FXS

Different mechanisms have been proposed to explain RS and its disruption in FXS. Simple, passive listening paradigms with short ISIs are designed to assess RS mediated through refractory properties of the neuronal network [9, 57]. The refractory system in FXS may be impaired through less synchronized and more widely excitable local synaptic networks due to exaggerated long-term depression found in *FMR1* KO mice [2] leading to weakened connections in neuronal circuits [9].

Another more cognitive theory accounting for RS is the sharpening model, proposing that repeated information leads to a ‘sharpening’ of information presentation in the cortex [13, 58–60]. While novel stimuli activate large non-specific populations of neurons, repeated stimuli exposure results in fewer firing neurons, with the response of these few neurons being more specific and thus sharper. The ‘predictive coding’ model is a neural network model [61] that explains sharpening through an interplay between bottom-up sensory input and top-down expectations in hierarchically organized sensory systems, ranging from the primary areas receiving sensory information from thalamic nerve projections to the frontal cortex generating a predictive percept [62]. RS is thereby the physiological correlate of a reduction in prediction error in response to a repeatedly presented stimulus that is achieved by modifying connections between hierarchical levels through synaptic plasticity [63]. Four phenomena identified in *FMR1* KO mice, closely entwined with deficient synaptic plasticity, might be involved in disrupted RS with regard to the sharpening theory: (1) hyperexcitable neurons [30], in interplay with (2) delayed and weaker inhibition [64], (3) less sharply selective neurons [30] with broader frequency-tuning curves [31] and, finally, (4) abnormal dendrite morphology that is closely related to defects in circuit plasticity [65]. Long dendritic spines with immature morphologies and higher spine density suggest a failure in the synapse maturation process [66, 67]. Recently, an interest was developed regarding synaptic BK channels that are crucial for short-term habituation and directly interact with FMRP [31, 68–71]. The BK channel seems to be involved in the abnormal dendritic spine phenotype [69], learning deficits [70] and hyperexcitabilty in FXS [71]. These mechanisms might be less affected in less severe ID FXS subgroups, since they are expected to have higher levels of FMRP.

Another factor that might mediate NVIQ-related differences in RS is attention, since the > 42 NVIQ group can be expected to pay more attention to the auditory stimulation. Attention-based prediction of up-coming stimuli modulated by the ventral striatum and the prefrontal cortex are central in the predictive coding model of RS [72, 73]. Concordantly, our group showed through transcranial direct current stimulation (tDCS) over the dorsolateral prefrontal cortex (DLPC) an enhancement of RS when the DLPC was excited and a reduction of RS when the DLPC was inhibited [40, 74]. Dorsolateral caudate circuitry has been found abnormal and related to cognitive and behavioural deficits in FXS [75]. Recent reports highlight the contribution of hippocampal memory activity in predictive coding [76–78], whereas a larger hippocampal size has been associated with worse memory in FXS [79].

Lastly, recent studies by Van der Molen et al. and Wang et al. have suggested that the lack of RS in FXS might be the result of uncoordinated neuronal synchronization patterns, since an imbalance between slow and fast oscillatory activity has been found in FXS [80, 81]. Elevated baseline levels of gamma power in FXS were interpreted as increased ‘background neural noise’ that contributed to impairments in synchronizing gamma frequency activity when necessary, leading to hyperexcitable and disorganized cortical networks [9]. These results underline the importance of comparing evoked responses against baseline levels in order to differentiate them from high neural background noise. Consequently, we normalized the time series of each presentation of a stimulus in our energy measures and examined the non-phase locked energy response relative to all ten presentations (see the ‘Methods’ section).

### Main factors of spatial PCA

Given that previous EEG studies found different scalp distributions of ERPs in FXS and controls [13, 15, 17, 18], we conducted a separate spatial PCA analysis for FXS and controls in order to identify all ROIs relevant for the performed task in our study population. Interestingly, the spatial PCA differed not only in location, but also in number of factors. The concentration of activity in the central and frontal areas in FXS is in line with what has been found in previous studies which found a more frontal distribution of AEPs [13] and higher AEP/ERP amplitudes over central electrodes in FXS [15], whereas no differences were found over the posterior and occipital sites when compared to controls [18]. This focus of auditory hyperexcitability over fronto-central sites in FXS might contribute to the fact that these two factors (central, left frontal) explain a major part of the data variance whereas spatial components of activity in the control group appears more distributed and complex, having a total of five factors explaining the majority of the data variance. Further, altered functional connectivity and brain network activation has been found in FXS, with increased spatial spreading of phase synchronised activity, which may account for a more unitary activation pattern [80, 81].

### Pseudoword learning and NVIQ

Language is typically a major deficit in FXS individuals, although receptive vocabulary is described as a relative strength in their cognitive domain [82]. In this study, cognitive functioning of FXS participants was evaluated using the non-verbal Leiter-R since it eliminates language deficit confounds. Furthermore, it can estimate NVIQ as low as 30, whereas a floor effect would have been expected when using most other batteries. The fact that repetition effects in response to pseudowords are predictors of NVIQ may suggest that repetition effects are of core importance for cognitive development, independent of the modality being investigated. As such, alterations of the repetition effects have been found in not only the auditory but also the visual modality [22, 23].

### Conflation between sex and NVIQ effects in FXS

Sex is an important confounding variable when investigating cognitive functioning in FXS. Given that females have a milder phenotype than males with FXS [3], all of our female FXS participants fell into the > 42 NVIQ subgroup and we did not have not enough statistical power to compare RS between male and female participants in this subgroup. Given the fundamental biological differences between males and females presenting FXS, such as but not limited to FMRP levels, our study may have shown differences in RS as much in relation to sex as to NVIQ. Our results show that the more cognitively affected a FXS participant is, the less likely they are to show RS. Female FXS participants, who are generally less affected, are more likely to show some RS, although delayed.

### Limitations and perspectives

Due to difficulties inherent to rare disease studies, our sample size is small, especially in the exploratory analysis in the median-split subgroups. Thus, single-trial machine learning was added in order to verify if our results would be confirmed with a completely different analysis approach. Studies investigating EEG in FXS often have small sample sizes, since difficulties in EEG recordings are a common problem [83]. These problems result in selection bias, since participants with severe ID and intense behavioural problems can rarely be tested, and as such, we are not able to investigate the full spectrum of FXS. Less invasive EEG setups, such as wireless nets and home recordings, may enable an adaptation of study procedures for this population. In this study, we demonstrated that reliable results could be obtained with a very short test (5 min), supporting the feasibility of EEG for FXS participants in a clinical setting.

We included a rather large age range (9–32 years) to avoid further reducing the sample size. Electrophysiological activity is known to change with age, and maturation effects might present a confounding variable in our analysis. Also, RS effects in specific AEP components are difficult to compare across age groups since morphology, amplitudes and latencies change with maturation [84]. Signal energy is less affected by maturational changes found in AEP components, since it summarizes all amplitude values within a given time window and thus allows for a global examination of repetition effects across presentations, independent of specific AEP components [40]. Further, the LMM approach can take variations in intercept and slope between participants into account. When entered as a possible predictor in our LMM, age did not significantly improve model fit, suggesting that age did not contribute to the explanation of repetition effects. Lastly, we controlled for age by using a control group with a similar age distribution.

Similarly, FXS participants in the ≤ 42 NVIQ FXS subgroup showed more autism symptoms, such as repetitive and aberrant behaviour, even though no statistical differences were found due to a lack of power for multiple testing. Segments containing movement artifacts were removed for all participants, and no significant difference for segments kept was found between FXS subgroups. As mentioned above, attention deficits in FXS participants could have perhaps disrupted RS. Although both subgroups present ADHD comorbidities, the severity of attention deficits may differ between groups.

A study evaluating the effect of these confounding variables would require a large *N* of different ID and autism populations as well as enough variability on all the variables to match and compare participants. This could most realistically be done in a multi-centric setting. Further, FMRP levels of FXS participants would have been of interest, since they likely represent a mediating factor between underlying neuronal alterations and severity of cognitive, behavioural and RS deficits.

### Medication

The majority of our FXS population was medicated with different psychoactive drugs. Since psychoactive drugs are known to influence parameters of electrophysiological activity, it is possible that drug effects are masking or creating effects found in our sample. Type of medication and dosage differed between all FXS participants, rendering a detailed examination of drug effects difficult due to small sample sizes in each subgroup. However, both FXS NVIQ subgroups contain a comparable amount of medicated and non-medicated individuals (see Table 2), suggesting that neither of the effects found in either group can solely be attributed to medication.

### Meaning of the median-split

It is important to underline that the median-split at a NVIQ of 42 was used as a statistical tool in order to explore the interaction between group, NVIQ and repetition effects revealed by the LMM. Repetition effects appeared to differ relative to NVIQ, but further analyses were necessary to get an idea of how patterns of RS change with NVIQ. Given the small sample size, a median-split was chosen, in order to have a similar *N* in each subgroup. Since two individuals had an NVIQ of 42, they were both included in the ≤ 42 subgroup, whereas the lowest NVIQ in the > 42 group has an NVIQ of 52, rendering the separation between both groups to ten NVIQ points. However, this artificial NVIQ cut point is not clinically meaningful. Considering that NVIQ co-varies significantly with RS in FXS, it is to be expected that participants further away from the split-point present a better model fit than participants close to a NVIQ of 42/52 who are more likely to be located somewhere between the two patterns explored in the median-split analyses.

## Conclusion

Due to their sensitivity, EEG measures may be a promising treatment biomarker. One important asset of such a biomarker is its independence from task comprehension, which is inherent to classic cognitive testing. Further clinical trials are needed to demonstrate direct treatment effects on cognition. We propose presentation-by-presentation EEG repetition effects as a sensitive tool in order to display modifications in brain processes relevant to cognitive and behavioural development in FXS.

## Additional file

Additional file 1: Supplementary methods and results section (DOCX 22 kb)

## Abbreviations

AEP: Auditory evoked potential
ASD: Autism spectrum disorder
C: Central ROI
CHU: University Hospital Center
DA: Decoding accuracy
DLPC: Dorsolateral prefrontal cortex
EEG: Electroencephalography
ERP: Event-related potential
FC: Fronto-central ROI
*FMR1*: Fragile X mental retardation 1
fMRI: Functional magnetic resonance imaging
FMRP: Fragile X mental retardation protein
FR: Frontal-right ROI
FXS: Fragile X syndrome
ICA: Independent component analysis
ID: Intellectual disability
IQ: Intelligence quotient
KNN: *k*-th nearest neighbor
KO: Knockout
LDA: Linear discriminant analysis
LMM: Linear mixed model
mGluRs: Metabotropic glutamate receptors
MMN: Mismatch negativity
NVIQ: Non-verbal IQ
PCA: Principal component analysis
RE: Repetition enhancement
RF: Random forest
ROIs: Regions of interest
RS: Repetition suppression
SVM: Support vector machine
tDCS: Transcranial direct current stimulation
TL: Temporal left ROI
WASI: Wechsler Abbreviated Scale of Intelligence
