# Supplementary material for: Auditory repetition suppression alterations in relation to cognitive functioning in fragile X syndrome: a combined EEG and machine learning approach
Source: J Neurodev Disord. 2018 Jan 29;10:4. doi: 10.1186/s11689-018-9223-3 (PMC5789548; doi:10.1186/s11689-018-9223-3)
Supplement: Additional file 1: — Supplementary methods and results section (DOCX 22 kb) [file 11689_2018_9223_MOESM1_ESM.docx]

**Supplementary methods and results section**

**Methods**

**ICA**

Blink detection was performed using the Value Trigger algorithm to optimize the detection of the prototypical blink patterns. Infomax Extended Biased technique was used for the independent component analysis (ICA) decomposition (number of ICA steps: 512) [39]. Identification of ICA components related to vertical and horizontal eye movement activity in reference channels 8 and 25. The correlative score (sum of squares) between the component activation and the activity of the selected channels was used to identify and delete eye movement artefact components (60% total value to delete).

**Signal energy**

The normalized time series *Y(t)* of each trial (each presentation *p* of each stimulus *s*) is defined as:

$$Y_{s,p}(t)=\frac{{amp}_{s,p}(t)-\sum_{t=1}^{Nt} {amp}_{s,p}(t)}{\frac{1}{Np}\sum_{p}^{Np} std({amp}_{s,p})}$$

Where ${amp}_{s,p}$ is the amplitude of the data points *t* (1 to the number of data points *Nt*) contained in a segmented trial for each presentation *p* (1 to *Np*) and each stimulus *s*, and *std* is the standard deviation of the trial. Then, data points of each trial are re-centered and divided by the average standard deviation of the series of presentations it belongs to. In order to compute the average induced energy (*Eavg*) of each presentation *p,* the induced energy (*Eind*) of the normalized trial of each presentation *p* and each stimulus *s* were first computed:

$${Eind}_{s,p}=\sum_{t}^{Nt} {{|Y}_{s,p}(t)|}^{2}$$

The induced energy of each presentation *p* is averaged across stimuli *s*:

$${Eavg}_{p}=\frac{1}{N_{s}}\sum_{s}^{Ns} E_{s,p}$$

**PCA**

Each spatial factor represents a specific spatial configuration of brain activity and the factor loading corresponds to the spatial factor’s contribution to the original variables (i.e. how much the spatial factor accounts for the voltage recorded at each electrode). These spatial configurations are defined by considering electrodes with the highest loading factors [43] and visualized by topographic maps of loading factors (Cartool software v.3.52, D. Brunet, https://sites.google.com/site/fbmlab/cartool). Here, a group of electrodes was identified as a region of interest (ROI) when the loadings of these electrodes were superior to 0.7, corresponding to more than 50% of the data variance being explained.

**Linear mixed models**

The model used for this study was determined by a series of steps to determine model fit [47], starting with evaluating if random intercept and random slope would improve the baseline repetitions*ROI model according to Chi-square likelihood ratio test. Then, the appropriate polynomial structure for changes in energy across repetitions was chosen by comparing model fit for linear, quadratic and cubic structures [49].

**Results**

**Baseline model: intercept, slope and polynomial structure**

The construction of the model was started with a simple repeated measures (repetition (10) * ROI (7)) model with energy as outcome variable, repetition and ROI as fixed effects and without any predictors that served as baseline model to examine if a random intercept is appropriate. The Chi-square likelihood ratio test displayed a better fit without as compared to with random intercept [χ^2^ (1, N = 40) = 11, p < 0.001]. Thus, considering participants’ random initial response to the first presentation of a pseudoword did not improve the model [47]. Next, we introduced a random slope to the model, allowing slopes across repetitions to vary between participants, which significantly improved the model [χ^2^ (1, N = 40) = 146, p < 0.001]. In order to examine the fit of a quadratic curve model, we added a quadratic term for repetition (repetition*repetition) to the model, which displayed a significantly poorer fit compared to the linear model [χ^2^ (4, N = 40) = 707, p < 0.001]. Since a test for cubic polynomial structure is only recommended when the quadratic structure improves the model [47], we continued with a linear curve model, which displayed the best fit.

**Baseline model: controls**

A baseline model without random intercept [vs. with random intercept: χ^2^ (1, N = 26) = 75, p < 0.001], with random slope [vs. without random slope: χ^2^ (1, N = 26) = 140, p < 0.001], linear structure [vs. quadratic χ^2^ (7, N = 26) = 422, p < 0.001] and first-order autoregressive covariance structure presented the best fit for the control data.

**Baseline model: FXS**

As for the baseline model, a random intercept diminished model fit for the FXS group [χ^2^ (1, N = 14) = 55, p < 0.001], whereas a random slope improved it [χ^2^ (1, N = 14) = 67, p < 0.001]. A quadratic polynomial presented significantly poorer fit than the linear model [χ^2^ (7, N = 14) = 339, p < 0.001]. First-order autoregressive covariance structure provided the best model fit.

**Baseline model: > 42 NVIQ FXS sub-group**

Best fit was observed for a baseline model without random intercept [vs. with random intercept: χ^2^ (1, N = 7) = 41, p < 0.001], with random slope [vs. without random slope: χ^2^ (1, N = 7) = 33, p < 0.001], linear structure [vs. quadratic [χ^2^ (7, N = 7) = 162, p < 0.001] and first-order autoregressive covariance structure.

**Baseline model: ≤ 42 NVIQ FXS sub-group**

A model without random intercept [vs. with random intercept: χ^2^ (1, N = 7) = 56, p > 0.001], with random slope [vs. without random slope: χ^2^ (1, N = 7) = 30, p < 0.001], linear structure [vs. quadratic [χ^2^ (7, N = 7) = 212, p < 0.001] and first-order autoregressive covariance structure presented the best fit for the data in the ≤ 42 NVIQ group.
